# Effect of age and sex on strength and spatial electromyography during knee extension

**DOI:** 10.1186/s40101-020-00219-9

**Published:** 2020-04-15

**Authors:** Ashirbad Pradhan, Gemma Malagon, Rebecca Lagacy, Victoria Chester, Usha Kuruganti

**Affiliations:** 1 Andrew and Marjorie McCain Human Performance Laboratory, Faculty of Kinesiology, University of New Brunswick, Fredericton, NB E3B 5A3 Canada; 2 Tecnologico de Monterrey, Monterrey, Mexico

**Keywords:** High density electromyography, Surface electromyography, Spatial distribution, Entropy, Aging, Vastus lateralis, Isokinetic knee extension, Isometric knee extension

## Abstract

**Background:**

Multichannel surface electromyography (EMG) is a method to examine properties of motor unit (MU) activity using multiple electrodes arranged on a two-dimensional grid. This technique can be used to examine alterations in EMG activity distribution due to contraction intensity as well as due to physiological differences such as age or sex. Therefore, the purpose of this study was to compare strength and high-density surface EMG (HDsEMG) features during isometric and isokinetic knee extensions between older and younger men and women.

**Methods:**

Twenty younger (ages 19–25 years) and twenty older (ages 64–78) men and women performed submaximal and maximal isometric (at a joint angle of 90°) and isokinetic knee extensions, while HDsEMG was recorded from the vastus lateralis. Spatial distribution was estimated using the root mean square (RMS), and 2-dimensional (2D) maps were developed to examine spatial features. Coefficient of variation (CV) and modified entropy were used to examine alterations in muscle heterogeneity and pattern. Peak torque and HDsEMG parameters were compared across age and gender.

**Results:**

Younger males and females produced significantly higher mean torque than the older group (*p* < 0.001) for all contractions. Both age- and sex-related significant differences (*p* < 0.05) were found for EMG spatial features suggesting neuromuscular differences. Modified entropy was significantly higher and CV was lower for young females compared to young males (*p* < 0.05) across both isometric and isokinetic contractions.

**Conclusions:**

We found that isometric and isokinetic knee extension strength, spatial distribution, and intensity differ as a function of age and sex during knee extensions. While there were no differences detected in entropy between age groups, there were sex-related differences in the younger age category. The lack of age-related differences in entropy was surprising given the known effects of aging on muscle fiber composition. However, it is often reported that muscle coactivation increases with age and this work was limited to the study of one muscle of the knee extensors (vastus lateralis) which should be addressed in future work. The findings suggest while both age and sex affect muscle activation, sex had a greater effect on heterogeneity. The results obtained will help to develop improved rehabilitation programs for aging men and women.

## Background

It is well established that aging leads to muscular weakness and morphological changes in skeletal muscle including a decline of muscle mass [1–3]. It has also been shown that strength declines in older individuals are greater in the lower than in the upper limb muscles [4]. These changes can cause movement deficiencies and compromise daily function with typical aging. Surface electromyography (EMG) employs electrodes placed over the skin to record the electrical activity (i.e., muscle action potentials) that brings about muscle contraction and provides important information regarding peripheral properties and central strategies of the neuromuscular system [4]. Surface EMG of skeletal muscle can be affected by numerous factors including contraction type, contraction intensity, and movement speed. In addition, the EMG is affected differently between males and females due to sex-related differences in myofiber composition as well as hormones [5], both of which can further compromise age-related factors. While intramuscular EMG can be used to examine age-related changes in motor unit (MU) function [6, 7], this technique is invasive and may be challenging for older populations. Surface EMG captures gross muscle activity rather than individual motor units (MUs) and can be used to evaluate muscle properties including those affected by age. More recently, the use of multiple two-dimensional surface electrode grids, or multichannel or “high density” EMG, have been used to evaluate muscle function successfully [8–12]. These high-density surface EMG (HDsEMG) recordings can be used to evaluate the detailed properties of MU activity [8]. The spatial distribution of multichannel surface EMG can be used to examine alterations in MU behavior [10, 13, 14]. Previous studies have used multichannel EMG to show that spatial activation distribution in a muscle is non-uniform and that the EMG spatial distribution pattern can be altered by contraction levels or fatigue [8–11]. In addition, multichannel EMG can be used to non-invasively investigate MU activation of muscle during force production at varying levels of force contractions [12]. Parameters and features of the spatial distribution pattern can be examined to further understand age- and sex-related differences in skeletal muscle. For example, MU loss and alteration in muscle fiber composition can result in “clustering” or large areas of muscle being occupied by similar types of muscle fibers [15–17]. This clustering of similar types of muscle fibers has been shown to result in low heterogeneity in older males compared to younger men [12]. With respect to sex differences in MU behavior, multichannel EMG has been used to show that females exhibit more varied motor unit recruitment compared to males during sustained low-intensity isometric contractions [18]. That study found that females demonstrated lower modified entropy and increased coefficient of variation (CV) compared to males indicating increased heterogeneity in the spatial distribution pattern. While multichannel surface EMG is an indirect method of assessing MU behavior, it can provide insight regarding MU activation patterns, does not require insertion of needles, and therefore is more tolerable.

Lower limb muscles such as the quadriceps are critical for locomotion and activities of daily living (e.g., bathing, dressing, toileting); age-related changes in MU behavior in these muscles remain unclear [12, 19]. In addition, while previous work has suggested that age-related strength loss is related to motor unit firing and/or recruitment properties [19], it was limited to isometric (stationary) contractions. It is important to examine dynamic contractions with varying joint angles to determine the impact on motor unit properties. Isokinetic dynamometry allows the measurement of dynamic contractions in a controlled speed and enables researchers to examine dynamic movement in a controlled environment.

The purpose of this study was to compare strength and spatial EMG features during isometric and isokinetic knee extension with varying contraction intensities between older and younger men and women. We hypothesized that the older adults would have lower strength and EMG amplitude and lower heterogeneity compared to younger individuals. We also examined both males and females to investigate any sex-related differences in strength and spatial EMG features. While it has previously been shown that there are sex differences in variances of the multichannel surface EMG distribution in the vastus lateralis during low-level contractions [18], it is important to examine this during moderate and high levels of contraction to determine if the differences remain with increasing force. We hypothesized that females would have lower strength and EMG amplitude compared to males and there would also be differences in heterogeneity with varying contraction levels compared to males.

## Methods

### Participants

Forty individuals participated in this study in four experimental groups, young males (*n* = 10, mean age = 21.1 ± 1.4 years), young females (*n* = 10, mean age = 21.9 ± 0.7 years), older males (*n* = 10, mean age = 70.4 ± 3.2 years), and older females (*n* = 10, mean age = 73.3 ± 3.1 years). The general characteristics of the participants are shown in Table 1. All participants were healthy and did not report any injury to the upper leg or knee joint. Prior to beginning the study, participants were given a detailed explanation of the procedure and gave written consent. The experimental procedures were approved by the University of New Brunswick Research Ethics Board.

**Table 1:** Characteristics of the elderly and young participants. Data are reported as mean ± standard deviation for age, height, weight, and body mass index (BMI)

	Elderly male (*n* = 10)	Elderly female (*n* = 10)	Young male (*n* = 10)	Young female (*n* = 10)
Age (years)	70.4 ± 3.2	73.3 ± 3.1	21.1 ± 1.4	21.9 ± 0.7
Height (cm)	167.1 ± 24.8	159.4 ± 5.5	174.8 ± 4.4	164.0 ± 5.0
Weight (kg)	91.5 ± 29.8	62.3 ± 6.8	78.1 ± 11.2	69.3 ± 10.8
BMI (kg/m^2^)	27.8 ± 4.2	24.6 ± 2.8	25.5 ± 3.3	25.8 ± 4.0

### Experimental design

**Fig. 1:**
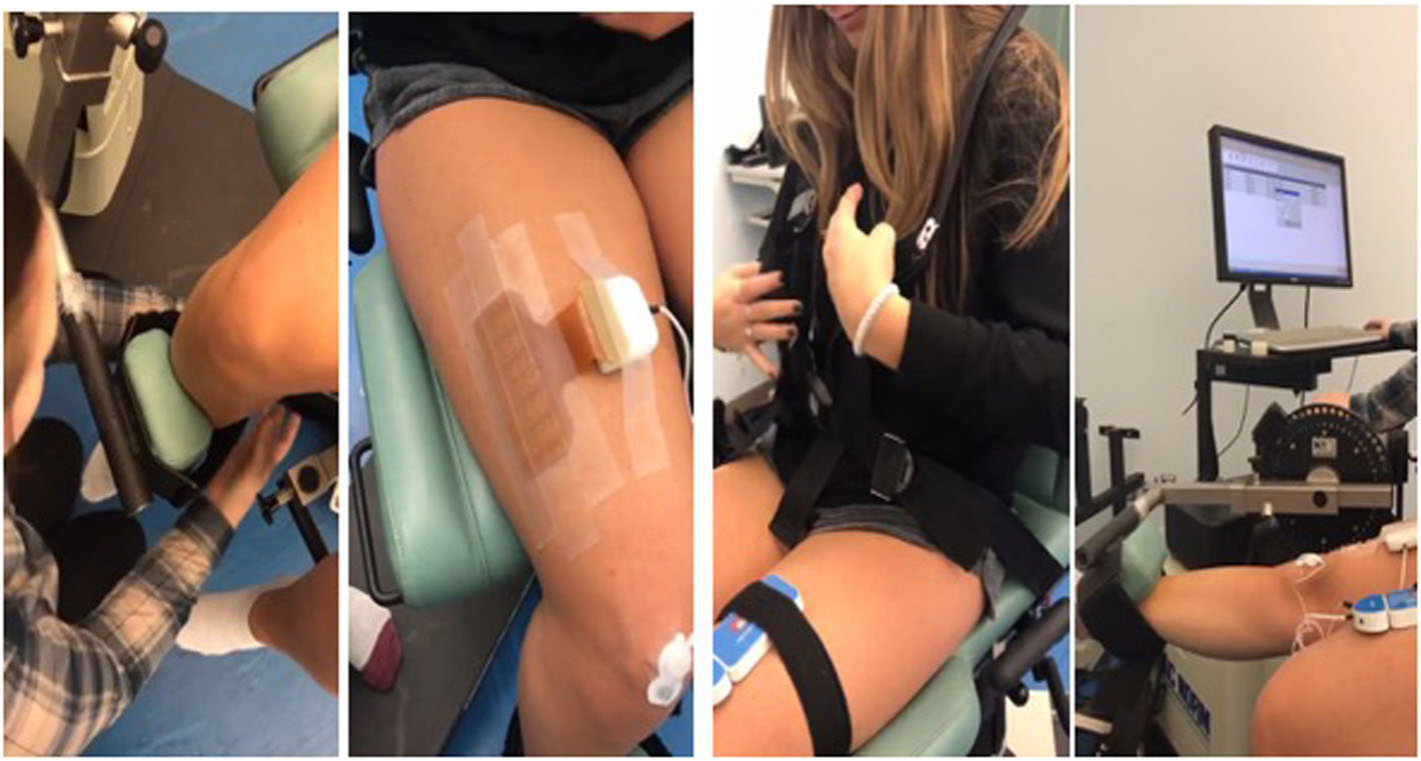
Experimental setup. The participant’s dominant leg was used for testing isometric and isokinetic knee extension

Participants were asked to perform a series of isokinetic and isometric knee extensions using a Cybex Humac Norm isokinetic dynamometer (CSMI Inc., USA). The isokinetic knee extensions were performed at a moderate speed of 1.05 rad/s, and the isometric contractions were completed with a knee angle of approximately 90° (measured with a goniometer) with the velocity of the dynamometer set to 0°/s (for the stationary contraction). Participants were seated on the dynamometer with their dominant leg strapped to the knee extension adapter approximately 2 in. above the ankle joint as shown in Fig. 1. Once the isokinetic dynamometer was adjusted to the individual, the participant was asked to keep their arms folded across their chest and begin the protocol with 3–5 practice contractions at a moderate level of effort. The participant was asked to fully extend and flex their knee during an isokinetic contraction to become familiar with the machine. There was a 3-min rest following the practice trial prior to beginning the testing protocol.

For the testing protocol, the participant was asked to complete three trials of an isokinetic knee extension at 1.05 rad/s. Throughout the contraction, the participant was provided visual feedback and verbal encouragement to “push as hard as you can.” Once the isokinetic contraction testing was complete, the participant was repositioned in the dynamometer chair and their leg was repositioned such that their knee angle was approximately at 90°. The velocity of the dynamometer was set to 0°/s for isometric testing and the participant was asked to perform at least two maximum voluntary contraction (MVC) trials with ≥ 5 min rest in between. The highest MVC torque was then used to calculate the target torque for the submaximal contractions (25, 50, and 75% of MVC). The participant was then asked to complete three isometric knee extensions at 25, 50, and 75% of their MVC. The isometric contractions were randomized and ≥ 5-min rest was provided between each contraction to avoid fatigue. From each contraction, the trial with the highest torque production was used for subsequent data analysis.

### Multichannel EMG recording

Multichannel high-density surface electromyography (HDsEMG) data were recorded during all contractions at 1024 Hz using a wireless high-density EMG (Sessantaquattro, OT Bioelettronica, Italy; input impedance, > 90 MΩ; CMMR, > 96 dB; filter, 10 Hz low cut-off; 500Hz high cut-off; noise, < 2 μV_RMS_ ) with a semi-disposable 64-channel electrode grid (ELSCH064NM2, OT Bioelettronica, Italy). The grid is comprised of 13 rows and 5 columns of electrodes. A double-sided adhesive foam grid was placed over the electrode grid, and conductive cream was inserted into each cavity to allow for skin contact to each electrode. The vastus lateralis of the participant’s dominant leg was palpated and the electrode grid placed over the muscle. Prior to electrode placement, the area was shaved to remove hairs, cleaned with an alcohol swab, and abraded with a paper towel. Additionally, a pre-gelled ground electrode (Duo-Trode, Myotronics Incorporated, Washington, USA; diameter, 12.5 mm; material, Ag/AgCl) was placed on the patella.

### Data analysis

HDsEMG data was recorded using specialized software (OT Biolab, Bioelettronica, Italy). Fifty-nine bipolar surface EMG signals along the columns were made from 64 electrodes. To calculate root mean square (RMS), EMG signals were analyzed using a 1-s interval centered at the midpoint of the contraction.

Spatial distribution was estimated using the RMS value for each of the electrode grid locations for each participant, and 2-dimensional (2D) maps were developed for each participant. The HDsEMG maps represent the spatial distribution of intensities of active MUs over the surface of the muscle as follows [20]:

HM_*ij*_ = RMS(sEMG_*ij*_)

where HM is an activation map and each pixel in a map (HM_*i,j*_) corresponds to an RMS value of a channel in an electrode array (position *i,j*).

It has been suggested previously that the relationship between EMG amplitude and force generated is not linear [2, 21], and therefore intensity was defined similar to previous work [20, 22] as the common logarithm of the mean intensity of the HDsEMG maps:

$$ I={\log}_{10}\frac{1}{N}\sum \limits_{i,j}\mathrm{H}{\mathrm{M}}_{i,j} $$

where *I* is an intensity feature calculated from the HDsEMG intensity map HM with a total number of *N* channels, and HM_*i,j*_ is the intensity of a channel located at position *i*,*j*.

The intensity of a single differential channel or differential intensity (DI) was calculated as a common logarithm of an RMS value of difference of two consecutive channels in the direction of the muscle fibers [20]:

$$ \mathrm{DI}={\log}_{10}\left(\mathrm{RMS}\left({\mathrm{sEMG}}_{i,j}-{\mathrm{sEMG}}_{\mathrm{i}+1,j}\right)\right) $$

Modified entropy and coefficient of variation were used to characterize the heterogeneity in the spatial multichannel HDsEMG potential distribution similar to previous studies [8, 12, 18]. Modified entropy of the spatial distribution of the EMG amplitude was calculated for 59 RMS values (in space) of single differential signals computed over a 1-s epoch during the contractions.

Maximum entropy occurs when all of the channels have the same RMS (log_2_59 = 5.884), and minimum value occurs when all of the channels are 0 except for one. Therefore, an increase in entropy indicates a decrease of heterogeneity of muscle fiber types, since muscle fibers innervated by the same motor neuron will have similar EMG recordings. Lower heterogeneity is expected in older adults due to the clustering of muscle fibers. Modified entropy was defined as the entropy of the signal power similar to Farina et al. [8]:

$$ E={\sum}_{i=1}^{59}p{(i)}^2\ {\log}_2\ p{(i)}^2 $$

where *p*(*i*) is the square of the RMS value of channel *i* divided by the sum of the squares of all 59 RMS values; therefore, *p*(*i*)^2^ represents the normalized power of each channel.

CV was calculated as the standard deviation (SD) of the 59 RMS values divided by the average of the 59 RMS values. When the SD is small relative to the mean, there is a smaller CV. Therefore, when the channels are more uniform, there will be a small CV, which also indicates homogeneity (low heterogeneity) of muscle fibers.

The recorded signals were analyzed off-line using the custom-built open-source MATLAB software HDsEMG Analysis Tool (Version 1.0; https://github.com/ashirbadpradhan/HDsEMG-Analysis-Tool-1.0). The following variables were computed to compare the group data: intensity, differential intensity, modified entropy, and coefficient of variation.

### Statistical analysis

All data are presented as the mean and SD for each group. Prior to beginning analysis, the normal distribution of the data was confirmed using Shapiro-Wilk’s test. The parametric analysis was used for normally distributed data, and the non-parametric analysis was used for non-normally distributed data. Torque, CV, intensity, differential intensity, and modified entropy were analyzed using two-way (age and sex) analysis of variance (ANOVA) with contraction level as a within factor. In case of a significant interaction between age and sex, a focused analysis was performed for each contraction level by fixing one of the between factors (sex and age) and comparing the other. The post hoc analysis was performed using Bonferroni correction method. RMS features were compared using Mann-Whitney test. All of the statistical tests were performed using RStudio 1.0. 136 (RStudio, Boston, MA).

## Results

**Fig. 2:**
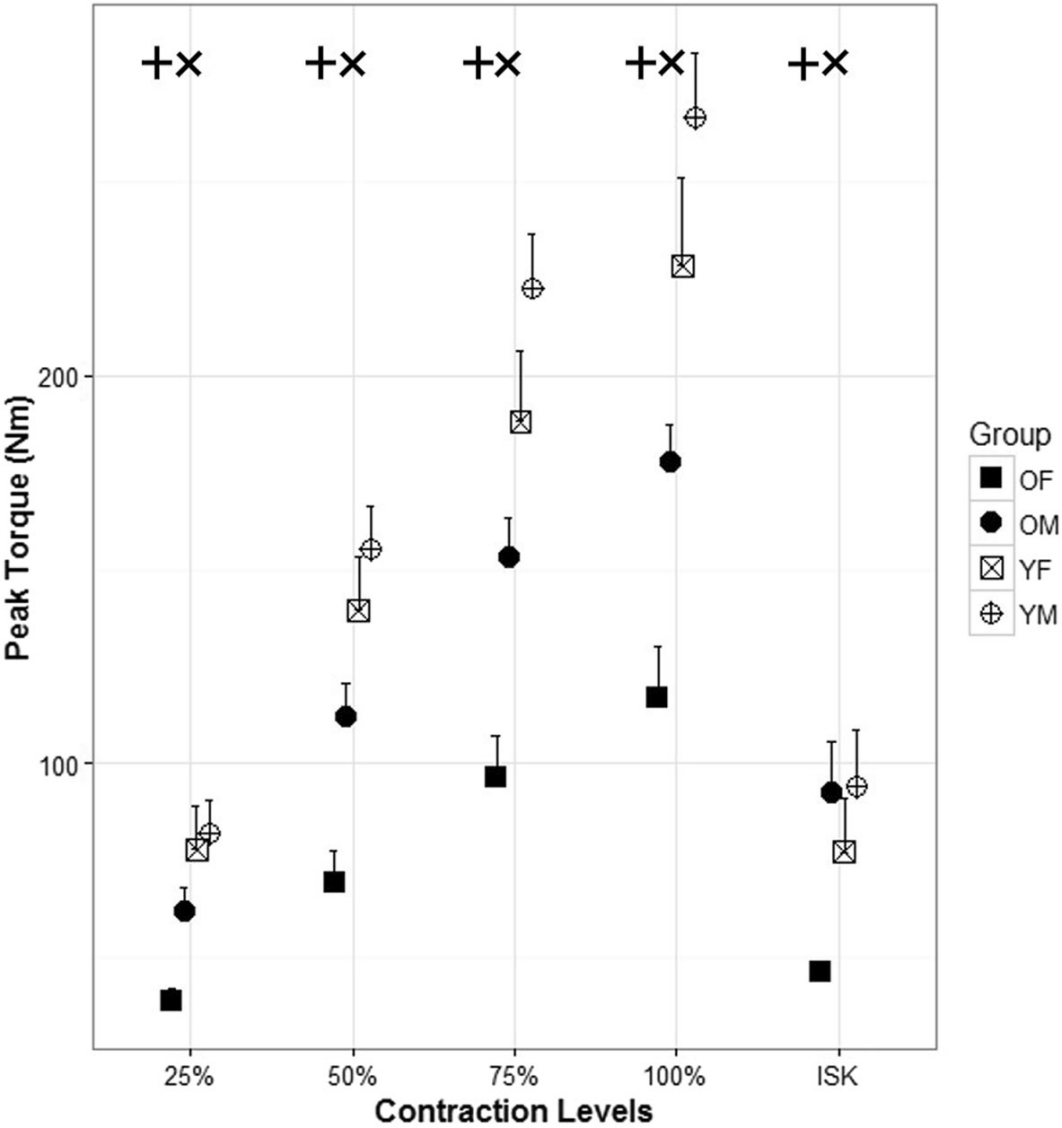
Peak torque (Nm) for each level of isometric contraction for each goup, older females (OF), older males (OM), younger females (YF) and younger males (YM)

The strength data showed age and sex differences consistently across contraction levels (Fig. 2). Older males produced significantly higher torque than older females for the 25% isometric MVC (*p* = 0.0034), 50% isometric MVC (*p* = 0.019), 75% isometric MVC (*p* = 0.001), and the 100% MVC (*p* = 0.0017). Similarly, older males had a higher isokinetic knee extension torque than older females (*p* = 0.0025). Significant differences were also detected due to age within each sex group. Younger females had significantly higher mean torque than the older female group for the isometric 25% MVC (*p* = 0.0035), 50% MVC (*p* < 0.001), 75% MVC (*p* < 0.001), and 100% MVC (*p* < 0.001). The younger females also produced significantly higher isokinetic extension (*p* = 0.037). The younger male group generally produced higher torque than the older male group with significant differences detected for the isometric 50% MVC (*p* = 0.012), 75% MVC (*p* = 0.011), and 100% MVC (*p* = 0.0017). Younger males produced higher but not significantly different strength than older males during the 25% MVC. There was no significant difference in isokinetic torque for knee extension between younger and older males.

**Fig. 3:**
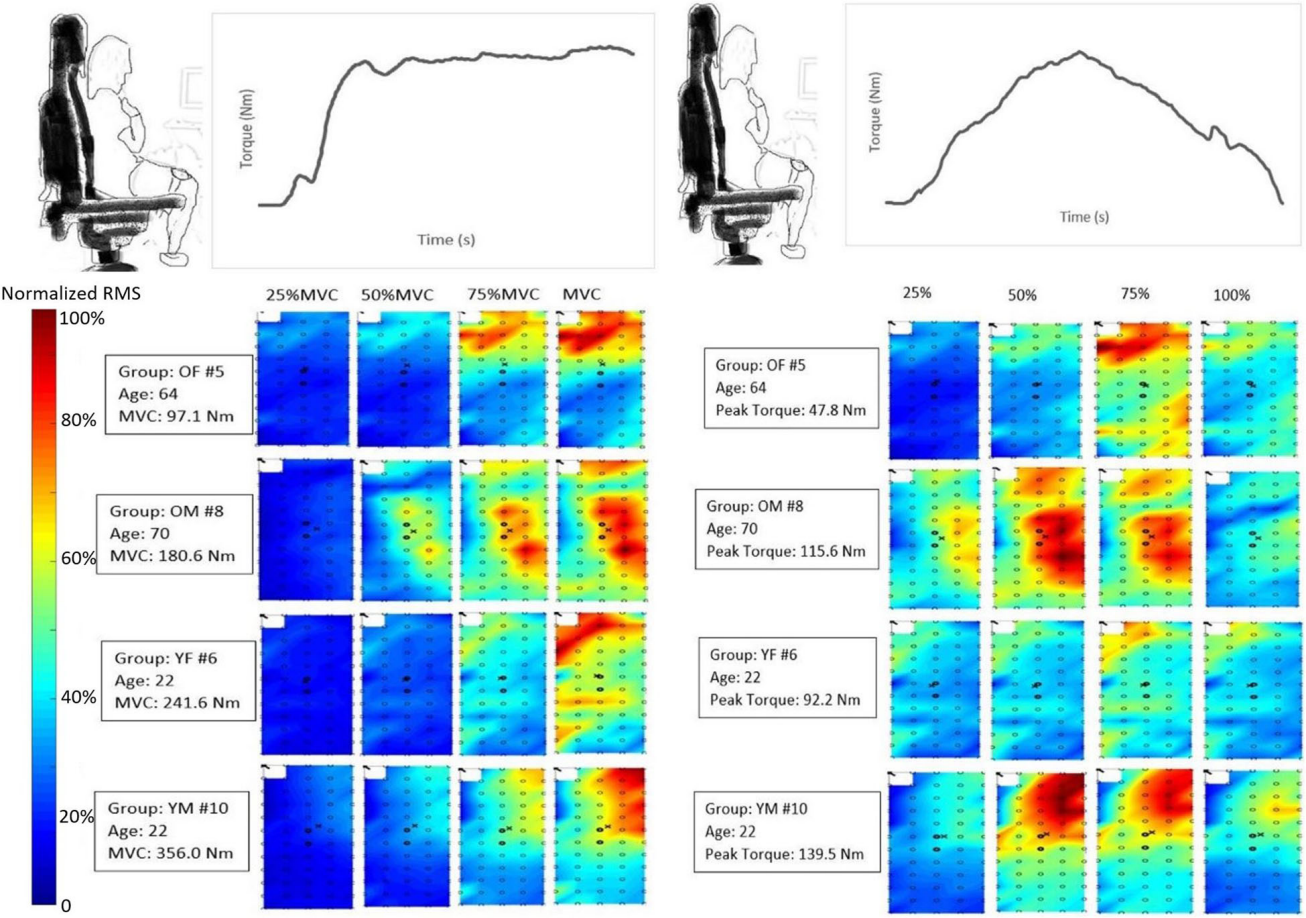
Sample color maps for isometric and isokinetic contractions. A sample color map from one participant from each tested group. Note that the maps in the left pane were generated during each submaximal and maximal isometric knee torque (25%MVC, 50% MVC, 75% MVC, and 100% MVC). The maps in the right pane were generated for the isokinetic knee extension at four points of the overall muscle contraction cycle (25, 50, 75, and 100%). Peak knee extension torque at the midway point throughout the contraction. Dark blue indicates areas of low muscle activity and dark red indicates areas of high muscle activity

**Fig. 4:**
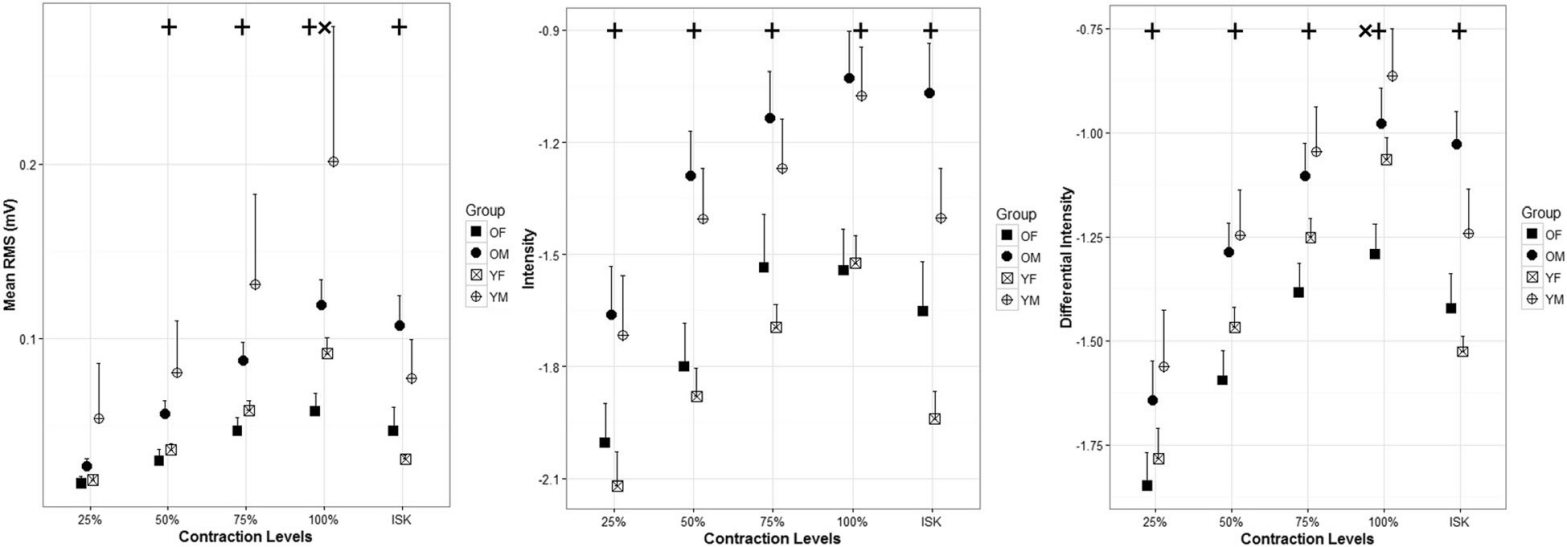
EMG spatial features. The first pane indicates the mean root mean square (RMS) values for each level of isometric contraction (25%, 50%, 75%, and 100%) and the isokinetic (ISK) contraction. The second pane indicates the intensity for each level of isometric contraction and the ISK contraction. The third pane indicates the differential intensity for each level of isometric contraction and the ISK contraction

Color maps were generated for each participant, and Fig. 3 illustrates data from sample participants for isometric and isokinetic (ISK) contractions (each map is scaled to the individual’s MVC). The color deviations in Fig. 3 range from dark blue to dark red indicating low to high levels of muscle activation. Mean and standard deviation for the HDsEMG variables including RMS, intensity, and differential intensity are presented in Fig. 4. An age effect was detected for the 100% MVC contraction, only with young females having a higher mean RMS than older females (*p* = 0.045); however, young males showed no differences in mean RMS compared to older males (*p* = 0.31).

Overall, males had significantly higher RMS for the 100% MVC compared to females (*p* = 0.049) as well as for the isokinetic knee extensions (*p* = .008). It was also found that older males had a significantly higher mean RMS than older females during the 50% MVC (*p* = .015), 75% MVC (*p* = .015), and 100% MVC (*p* = .05) isometric knee extensions. Overall, males (older and younger) had higher mean RMS than females (older and younger). Older males had significantly higher mean RMS than older females (*p* = .008) and younger males had a higher mean RMS than younger females (*p* = .023) for the isokinetic contraction.

Intensity showed differences as a function of sex depending on the contraction level (Fig. 4). There were no significant differences detected in intensity due to age; however, there were significant differences detected due to sex. The intensity was higher (i.e., less negative) for older males compared to older females for the 50% MVC (*p* = .007) and 100% MVC (*p* = .007) isometric knee extension and approaching significance for the 75% MVC (*p* = 0.051). Younger males had greater intensity compared to younger females at 25% MVC (*p* = .043), 50% MVC (*p* = .007), 75% MVC (*p* = .017), and 100% MVC (*p* = 0.008). For the isokinetic contraction, older males had significantly higher mean intensity compared to older females (*p* = .003).

Differential intensity showed significant differences due to age and sex depending on the contraction level (Fig. 4). Younger females demonstrated significantly greater differential intensity than older females (*p* = .02) for the 100% MVC. Older males had significantly greater differential intensity than older females for the 50% MVC (*p* = .007), 75% MVC (*p* = .022), and 100% MVC (*p* = .012) isometric knee extensions. Similarly, for the isokinetic contraction, older males had significantly higher mean differential intensity compared to older females (*p* = .003) and younger males had significantly higher mean differential intensity compared to younger females (*p* < .001).

A Pearson product-moment correlation coefficient was computed to assess the relationship between the mean HDsEMG RMS and peak torque. It was found that mean HDsEMG RMS amplitude was positively correlated with muscle strength and showed good to strong positive correlation for each age and sex group (Table 2).

**Table 2:** Pearson product-moment correlation between mean RMS and peak torque. There was a significant positive correlation (*p* < 0.05) between mean RMS and peak torque for each group, older females (OF), older males (OM), younger females (YF), and younger males (YM)

Group	Correlation coefficient	***p*** value
OF	0.47	0.002
OM	0.80	< 0.001
YF	0.82	< 0.001
YM	0.46	0.003

**Fig. 5:**
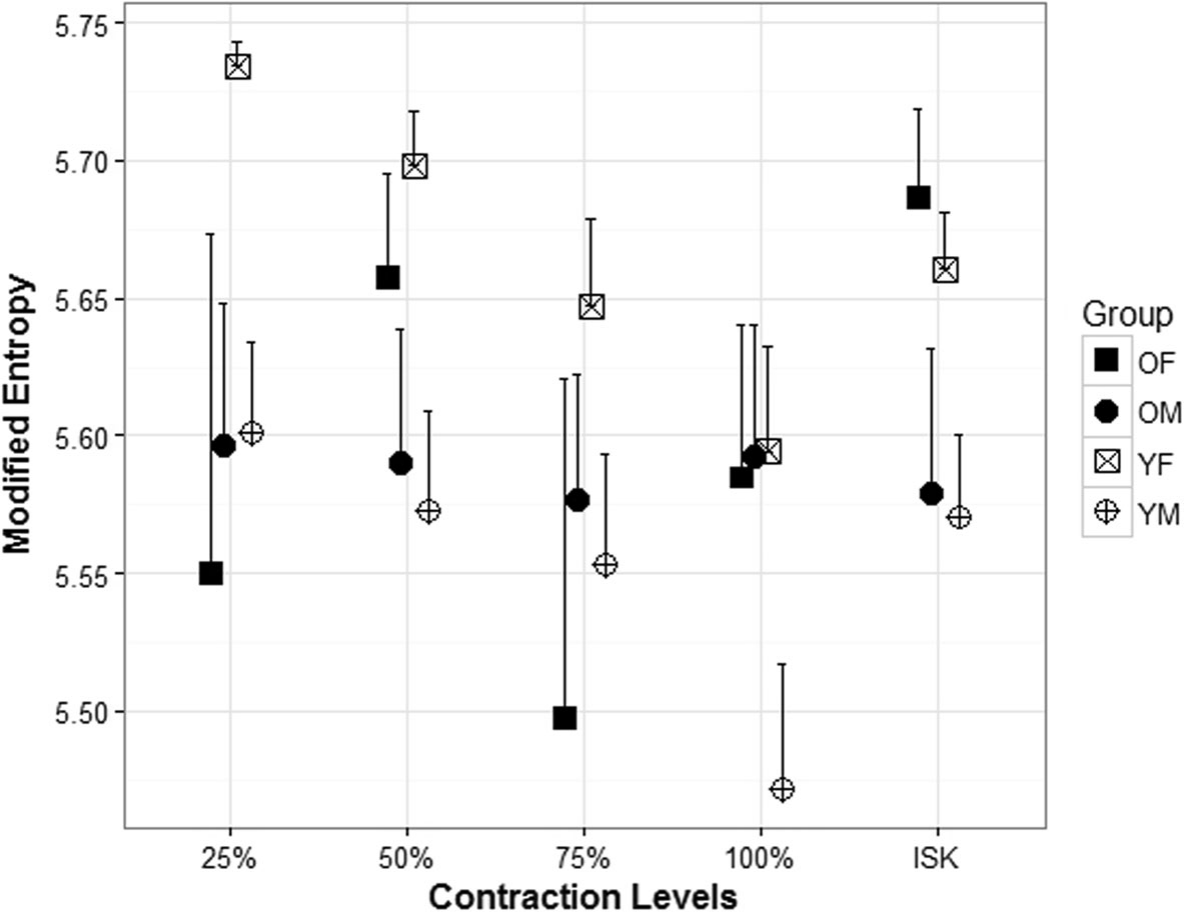
Modified entropy during submaximal and maximal isometric and isokinetic (ISK) knee extension. The data is shown for four groups, older females (OF), older males (OF), younger females (YF), and younger males (YM).

Modified entropy values across all groups for isometric and isokinetic contractions ranged from 5.47 to 5.73. While there were no differences detected in entropy as a function of age, there were sex-related differences in the younger age category (Fig. 5). The mean entropy during the 25% MVC isometric contractions was significantly higher (*p* = .01) for younger females (mean = 5.73 ± 0.03) compared to younger males (mean = 5.60 ± 0.11). Similarly, the mean entropy during the 50% MVC isometric contractions was significantly higher (*p* = .007) for younger females (mean = 5.70 ± 0.06) compared to younger males (mean = 5.57 ± 0.12). Finally, the mean entropy during the 75% MVC isometric contractions was significantly higher (*p* = .084) for younger females (mean = 5.65 ± 0.10) compared to younger males (mean = 5.55 ± 0.13). The mean entropy during the MVC isometric contractions was higher for younger females (mean = 5.59 ± 0.12) compared to younger males (mean = 5.47 ± 0.15), but the difference was non-significant (*p* = .05). The isokinetic contraction was similar with younger females exhibiting a significantly higher (*p* = .015) mean entropy (mean = 5.96 ± 0.02) compared to younger males (mean = 5.84 ± 0.14).

**Fig. 6:**
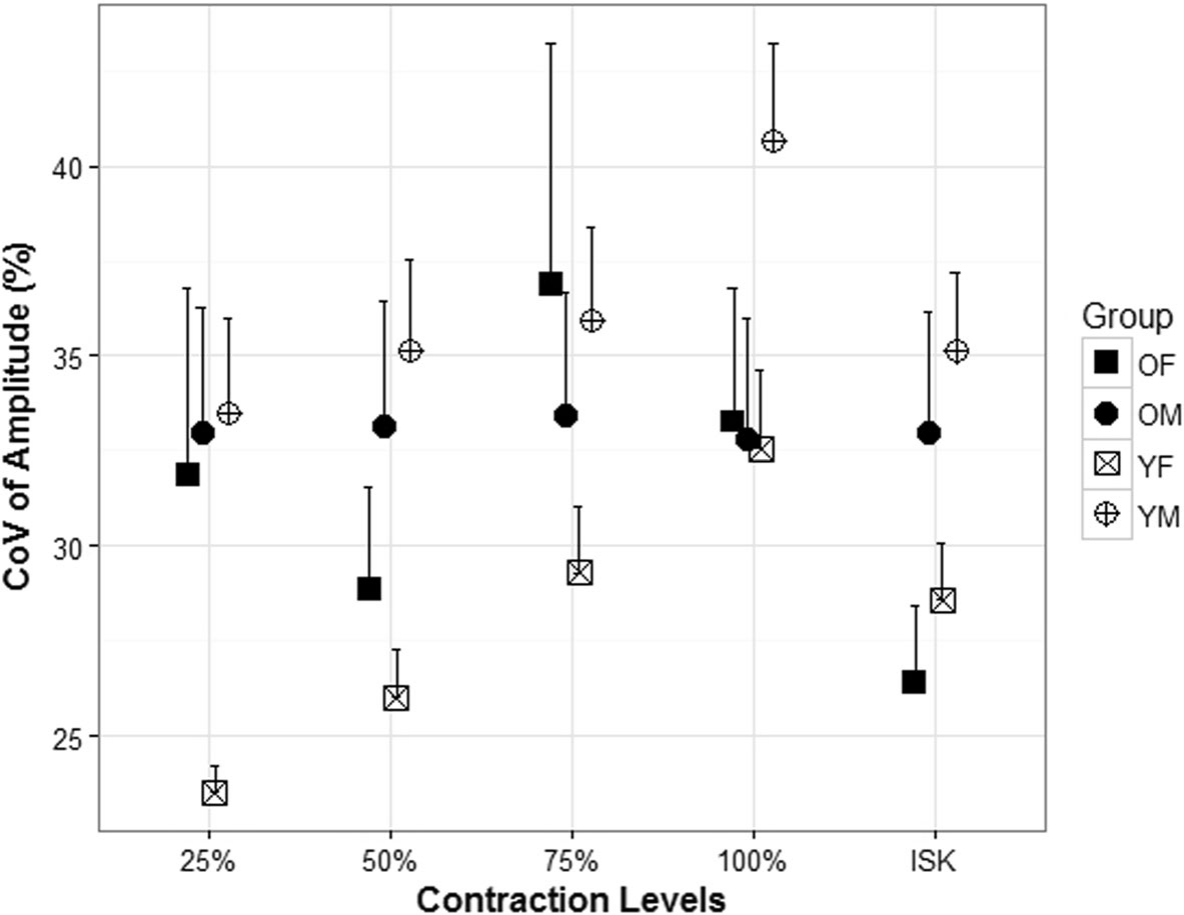
Coefficient of variation during submaximal and maximal isometric and isokinetic (ISK) knee extension. The data is shown for four groups, older females (OF), older males (OF), younger females (YF), and younger males (YM)

Similar to modified entropy, sex had a greater effect on CV than age (Fig. 6) but only in the younger groups. Younger males had a significantly higher mean CV for all contractions compared to younger females. The mean CV during the 25% MVC isometric contractions was significantly higher (*p* = .001) for younger males (mean = 33.50 ± 7.85) compared to younger females (mean = 23.50 ± 2.32). Similarly, the mean CV during the 50% MVC isometric contractions was significantly higher (*p* = .004) for younger males (35.16 ± 7.59) compared to younger females (mean = 26.02 ± 4.06). The mean CV during the 75% MVC isometric contractions was significantly higher (*p* = 0.04) for younger males (mean = 35.93 ± 7.69) compared to younger females (mean = 29.29 ± 5.58). Similarly, the mean CV during the 100% MVC isometric contractions was significantly higher (*p* = .024) for younger males (mean = 40.66 ± 8.14) compared to younger females (mean = 32.57 ± 6.46). Finally, the isokinetic contraction displayed similar sex-related differences with younger males having a significantly higher (*p* = .011) mean CV (mean = 23.31 ± 11.98) compared to younger females (mean = 12.11 ± 3.23).

## Discussion

This study examined strength and spatial EMG features during isometric and isokinetic knee extension between younger and older men and women. It was hypothesized that older individuals would exhibit lower strength and higher EMG amplitude as well as lower heterogeneity compared to younger adults. This study included an isokinetic knee extension which has not been studied previously. Age-related muscle loss and quality, or sarcopenia, is prevalent in older adults, and therefore it is not unexpected that strength was lower in those individuals. As expected, younger adults had significantly higher strength than older adults during both isometric and isokinetic knee extensions, with younger males producing the highest torques for both.

Unlike the strength data, age-related differences were not detected across all contraction levels in the HDsEMG data. However, sex differences were consistently noted in mean HDsEMG RMS suggesting differences in neuromuscular behavior. Other HDsEMG variables including intensity and differential intensity showed similar results suggesting that sex differences may have a stronger effect in muscle recruitment rather than strength. However, EMG was collected from only one muscle of the quadriceps (vastus lateralis), while the torque is the net force produced on the dynamometer and does not consider other contributing muscles (e.g., rectus femoris, vastus medialis) or antagonist muscle activity (i.e., from the hamstring muscles). Similar to previous research [23], we found that the RMS amplitude and torque were highly correlated for older males and younger females (Table 2). Interestingly, these results are similar to those found with conventional bipolar surface EMG techniques.

The color maps showed different patterns of activity for older adults compared to younger adults with older females displaying the greatest area of intensity throughout the isometric contractions (Fig. 3). The color maps from the isokinetic contraction (Fig. 3) showed the greatest activity between 50 and 75% of the contraction cycle which would align with the generation of maximal torque. It is important to note that the figures presented are representative samples from each age and sex group tested. In addition, the color maps may be different because of different locations of the innervation zone of the muscle.

Age-related differences were not detected in heterogeneity; however, there were sex-related changes that occurred in both entropy (Fig. 5) and CV (Fig. 6). Modified entropy and CV indicate heterogeneity in spatial HDsEMG distribution [12], and it has been suggested that heterogeneity in the EMG spatial distribution can be explained by a clustering of muscle fibers innervated by one MU in a restricted area [18] and that a decrease in entropy and increase in CV would suggest an increase in heterogeneity. The results of our study showed similar entropy and CV values to Watanabe et al. [12]. However, we did not detect any differences in entropy or CV with respect to increases in torque as they did. This could be due to the differences in the type of contractions between the studies. They noted a significantly smaller decrease in entropy and increase in CV with an increase of exerted torque in elderly and younger men. Furthermore, they found that modified entropy at 65% MVC (of ramped contraction) in the elderly participants was higher than younger individuals. In this study, we examined isometric knee extensions at 25, 50, 75, and 100% of the individual’s MVC. It has also been shown more recently [24] that interindividual differences in muscle strength for older individuals are related to the amplitude of the surface EMG and can affect multichannel surface EMG parameters which could partially explain why we did not see similar age-related differences in heterogeneity.

While we did not find changes due to age, we did note several significant differences in both entropy and CV due to sex (Figs. 5 and 6) during the isometric and isokinetic contractions. The differences detected between females and males may be due to differences in fiber type in the vastus lateralis. It has been reported that females have larger areas of type I fibers in the vastus lateralis compared to males [5, 25].

Nishikawa et al. [18] found significant differences in entropy and CV between males and females during a sustained low level (10% MVC) isometric knee extension. They found that females had lower modified entropy and increased CV suggesting increased heterogeneity in the spatial HDsEMG potential distribution resulting in differences in motor unit strategies between females and males during lower limb muscle activity with low-intensity sustained isometric contractions. Our results, unlike those of Nishikawa et al. [18], showed that younger females had significantly higher entropy than younger males during the submaximal isometric and isokinetic knee extension. However, the contraction intensity of the submaximal contractions in this study was higher than theirs and we also included maximal contractions. Considering Henneman’s size principle of motor unit recruitment, generally, type 1 fibers are primarily recruited during low-intensity contractions and could account for the different results. Furthermore, our findings suggest that sex-related differences occur over a range of isometric and isokinetic contractions.

It has been shown that there is a change in the spatial distribution patterns somewhere between 65 and 100% MVC in older populations caused by a change in the motor recruitment. De Luca and Hoastage [26] examined firing rates and motor neuron recruitment thresholds across three muscles, the vastus lateralis, first dorsal interosseous, and the tibialis anterior. By observing numerous motor units at high force levels in the vastus lateralis and the tibialis anterior (including MVCs), they observed that firing rates tend to group closer together as the force increases but also that they do not fully converge. They concluded that during isometric contractions, the control of motorneurons in the motorneuron pool maintains its structure among different muscles, there is variability among individuals. In the present work, we also examined an isokinetic knee extension in which the muscle contracts and shortens at a constant rate of speed which may affect heterogeneity. To our knowledge, this is the first study to examine spatial distributions changes including heterogeneity in this type of contraction. The modified entropy data showed little differences due to age in either males or females during isokinetic knee extensions. It is important to note that the isokinetic contractions were completed at one slow velocity (1.05 rad/s). It has been shown that, in older males, the greatest torque deficits in the knee extensors occurred at fast velocity [27, 28]. Future studies incorporating both slow and fast velocities may provide greater insight regarding velocity-dependent changes in torque and spatial distribution.

Some of the differences in our results compared to others may have been due to the anthropometric characteristics of the participants. One factor affecting surface EMG is the thickness of subcutaneous tissue. The amount of subcutaneous tissue between the originating EMG signal and the recording electrodes can act as a passive volume conductor which can determine the features of the detected signal. Unfortunately, we did not record participant’s physical activity levels nor muscle skinfold thickness prior to acceptance into the study which could account for some of the differences in entropy and CV in our study. As can be seen in Table 1, the BMI of the older males (mean = 27.8 ± 4.2 kg/m^2^) indicated that they were overweight compared to the other age and sex groups. While this may have had an impact on the results, it has also been shown that detection of surface EMG from the quadriceps is feasible even in severely obese individuals, that is those with a BMI greater than 40 [29]. It has also been shown that high-intensity physical activity can mitigate the age-related loss of motor units [24, 27]. Candow and Chilibeck [27] also found that torque and power as well as muscle thickness in the lower body are more affected by aging than upper body measures in men. The isometric knee extensions were completed at a knee joint angle of 90°; however, it has been observed that the highest knee extension torque occurs in the range of 115–140° [12, 30].

One limitation of this study is that it focused on measuring the EMG activity from the VL muscle, which is one of four muscles contributing to the strength of the quadriceps femoris. The lack of information regarding load in the knee extensor muscle should be addressed in future studies with larger grids as well as an increased number of grid locations for improved EMG-force correlations. We also did not record activity from the antagonist (hamstrings) muscle which may have affected overall strength production as well as neuromuscular activation, particularly in the older participants as muscle coactivation increases with age.

## Conclusion

In this study, we examined the strength and HDsEMG spatial features in both isometric and isokinetic knee extensions in younger and older men and women. While both age and sex differences were consistently detected in the torque data, the differences due to sex were more distinct in the HDsEMG features. This suggests that sex may have a greater effect on strength development and muscle spatial parameters than age alone. We also demonstrated that HDsEMG amplitude is positively correlated with muscle strength. Finally, while we found similar entropy values to previous research, our results indicated that sex had a greater effect on heterogeneity than age.

The color maps showed for both isometric and isokinetic contractions that older adults displayed greater areas of high intensity with higher force production. This was also reflected in the correlation between mean EMG amplitude and torque produced. This was the first study to examine isokinetic contractions which are more reflective of dynamic movement. Future work should examine isokinetic contractions during slow, moderate, and fast contractions as well as isometric contractions at varying joint angles to determine the impact on force development and spatial parameters. Aging muscle is physiologically different from younger muscle, and changes in spatial distribution may provide insight into muscle activation patterns and their variability provided that sex differences are accounted for.

## Data Availability

The datasets generated and/or analyzed during the current study are not publicly available due to privacy issues but are available from the corresponding author on reasonable request.

## Abbreviations

2D: Two-dimensional
ANOVA: Analysis of variance
BMI: Body mass index
CV: Coefficient of variation
DI: Differential intensity
EMG: Electromyography
HDsEMG: High-density surface electromyography
MU: Motor unit
MVC: Maximum voluntary contraction
RMS: Root mean square
SD: Standard deviation
VL: Vastus lateralis
